# An Investigation of the Indentation Elastic Modulus for Metal Films on Flexible Substrates Considering the Substrate Effect

**DOI:** 10.3390/ma18010154

**Published:** 2025-01-02

**Authors:** Jong-Hyup Lee, Ju-Been Ham, Young-Cheon Kim

**Affiliations:** Materials Research Centre for Energy and Clean Technology, School of Materials Science and Engineering, Andong National University, Andong 36729, Republic of Korea; 20225308@student.anu.ac.kr (J.-H.L.); 20245034@student.anu.ac.kr (J.-B.H.)

**Keywords:** flexible, mechanical properties, elastic modulus, nanoindentation

## Abstract

The accurate measurement of the elastic modulus of thin metal films on flexible substrates is critical for understanding the mechanical reliability of flexible electronics. However, conventional methods, such as the Oliver–Pharr model, often underestimate the modulus due to substrate effects, particularly with low-modulus substrates like polyimide (PI). In this study, we propose an improved weighting model that replaces the empirical weighting factor with a variable X to better account for substrate contributions. Nanoindentation experiments were performed on Cu and Al films with thicknesses of 0.5, 1, and 1.5 μm, deposited on PI and silicon substrates. The results show a significant underestimation of the elastic modulus when traditional methods were applied, especially on PI substrates, where values decreased by up to 95%. Using the proposed X-based model, the corrected elastic modulus aligned with the inherent properties of the films, with errors reduced to within 2%. A finite element analysis (FEA) validated the stress and displacement distributions, demonstrating the substrate’s influence on indentation behavior. This study provides a robust framework for accurately measuring the elastic modulus of thin films on flexible substrates, paving the way for a more reliable mechanical characterization in flexible electronics.

## 1. Introduction

Historically, electronic devices have relied on printed circuit boards (PCBs), which are rigid and difficult to deform, thereby limiting product design and portability. To overcome these limitations, ongoing research aims to develop flexible electronic devices by replacing traditional PCBs with more adaptable materials [1,2,3,4,5,6]. Among the most common flexible substrates used to replace rigid PCB materials are polyimide (PI) and polyethylene terephthalate (PET). The transition to flexible electronics has expanded the potential for innovative product designs, enabling devices that can bend, fold, and even roll [6,7,8,9,10,11]. This flexibility, while advantageous, introduces new mechanical challenges. Flexible electronics are often subjected to significant mechanical strain, which can lead to fatigue and eventual failure. In particular, foldable smartphones, which are gaining popularity, require high levels of mechanical reliability due to repeated mechanical stress at the hinge. To address this issue, ongoing research has focused on evaluating the mechanical reliability of thin metal films on flexible substrates under various conditions, such as torsional fatigue, reliability under rolling deformation, and fatigue life in extreme environments [12,13,14,15,16].

To ensure the durability of flexible electronics, it is essential to accurately assess the mechanical properties of the materials used. In contrast with conventional mechanical testing, nanoindentation is a relatively non-destructive testing method that offers a comprehensive evaluation of mechanical properties, such as hardness, elastic modulus, and residual stress, in a single test [17,18,19]. When assessing the mechanical performance of thin metal films on flexible substrates, the elastic modulus is a key property that must be accurately measured. Despite the widespread use of nanoindentation, accurately determining the elastic modulus of thin metal films on flexible substrates presents challenges to account for the influence of the underlying substrate, leading to an underestimation of the elastic modulus of the thin metal films. To address this limitation, several studies have proposed methods to reduce errors, such as correcting the contact geometry and separating the substrate and film in analyses [20,21,22,23].

In this study, we investigated the elastic modulus of thin metal films on flexible substrates through nanoindentation, focusing on the influence of substrate type and film thickness. Recognizing the limitations of conventional methods, which often underestimate the modulus due to substrate effects, we proposed an advanced correction method to improve measurement accuracy. Our approach involved evaluating the elastic modulus as a function of both the type of flexible substrate and the thickness of the metal film. Nanoindentation experiments were conducted using a 5 × 5 array, and reproducible data were selected for analysis. Thin aluminum (Al) and copper (Cu) films, with thicknesses of 0.5, 1, and 1.5 μm, were deposited on two different substrates: a 4-inch silicon (Si) wafer and 125 μm of polyimide (PI), known for its excellent mechanical properties. To address the challenges of accounting for substrate effects, we introduced a new weighting with a variable X, enabling a more accurate assessment of the substrate’s contribution to the measured elastic modulus. Additionally, a finite element analysis (FEA) was employed to verify the stress distribution during the indentation process, further validating the effectiveness of the proposed model.

## 2. Theoretical Background

The elastic modulus and hardness in nanoindentation tests are derived from analyzing the load–displacement curve. Key parameters such as the maximum indentation depth, load, and stiffness (S) can be obtained. Stiffness, corresponding to the initial slope of the unloading curve, is expressed as [24].

(1) $$s=\frac{dP}{dh}=\beta \frac{2}{\sqrt{\pi }}E_{r}\sqrt{A}$$

This equation is derived from Sneddon’s model, where *P* is the load, *h* is the displacement, *β* is a correction factor based on the indenter shape, *E_r_* is the equivalent modulus of elasticity, and *A* is the contact area.

Using Sneddon’s work as a basis, Bulychev et al. proposed the following equation to derive the equivalent elastic modulus in nanoindentation experiments [25].

(2) $$\frac{1}{E_{r}}=\frac{\left(1-{v_{i}}^{2}\right)}{E_{i}}+\frac{\left(1-{v_{s}}^{2}\right)}{E_{s}}$$

where *E_i_* and *ν_i_* are the elastic modulus and Poisson’s ratio of the indenter, and *E_s_* and *ν_s_* are the elastic modulus and Poisson’s ratio of the specimen.

Doener and Nix provide an empirical model for understanding the influence on thin film measurements during nanoindentation. Their model incorporates weighting factors that vary with indentation depth to the contributions of both the film and substrate [22].

(3) $$\frac{1}{E_{r}}=\frac{{1-v_{i}}^{2}}{E_{i}}+\frac{{1-v_{f}}^{2}}{E_{f}}\left(1-e^{-\frac{\alpha t}{h_{eff}}}\right)+\frac{{1-v_{s}}^{2}}{E_{s}}\left(e^{-\frac{\alpha t}{h_{eff}}}\right)$$

Here, *t* is the film thickness; *α* is an empirically determined constant; and the subscripts f, s, and i refer to the film, substrate, and indenter, respectively.

King refined this model by introducing the parameter a, representing the square root of the contact area, to account for cases where the film thickness is comparable to the indentation depth. King’s model is expressed as follows [23]:

(4) $$\frac{1}{E_{r}}=\frac{{1-v_{i}}^{2}}{E_{i}}+\frac{{1-v_{f}}^{2}}{E_{f}}\left(1-e^{-\frac{\alpha t}{a}}\right)+\frac{{1-v_{s}}^{2}}{E_{s}}\left(e^{-\frac{\alpha t}{a}}\right)$$

King’s model was further adapted for the Berkovich indenter by considering the indentation depth (*h*) and the effective film thickness t−h [26]:

(5) $$\frac{1}{E_{r}}=\frac{{1-v_{i}}^{2}}{E_{i}}+\frac{{1-v_{f}}^{2}}{E_{f}}\left(1-e^{-\frac{\alpha (t-h)}{a}}\right)+\frac{{1-v_{s}}^{2}}{E_{s}}\left(e^{-\frac{\alpha (t-h)}{a}}\right)$$

In this equation, t−h accounts for the film thickness minus the indentation depth. The parameter α is constant, varying with the indenter geometry and film thickness. This adaptation improves accuracy when the indentation depth approaches the film thickness by balancing contributions from both the film and substrate.

## 3. Experimental Section

**Figure 1:**
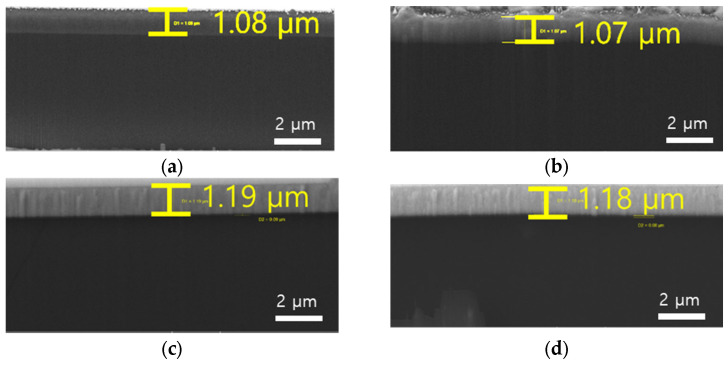
FIB images: (**a**) Al deposition on Si substrate, (**b**) Al deposition on PI substrate, (**c**) Cu deposition on Si substrate, and (**d**) Cu deposition on PI substrate.

Aluminum (Al) and Copper (Cu) films were deposited onto two types of substrates—125 μm-thick polyimide (PI) substrates and 4-inch silicon (Si) wafers—using sputtering. The films were deposited at thicknesses of 0.5, 1, and 1.5 μm to examine the impact of substrate type and film thickness on the elastic modulus. The deposition was carried out under a DC power of 50 W, with an argon (Ar) gas flow of 20 SCCM, and at a pressure of 1 mTorr. Deposition times were adjusted according to the desired film thickness: 77, 154, and 230 min for Al and 50, 100, and 150 min for Cu for thicknesses of 0.5, 1, and 1.5 μm, respectively. The thickness of the deposited Al and Cu layers has been confirmed using FIB, as shown in Figure 1.

Nanoindentation tests were conducted using Nano indenter (NanoFlip Nano indenter, KLA, Oak Ridge, TN, USA) system, equipped with a Berkovich indenter (Micro Star Technologies, Huntsville, TX, USA) that has an elastic modulus of 1140 GPa and a Poisson’s ratio of 0.07. To prepare the samples, an aluminum puck was first heated on a hot plate, and a glass plate was bonded to the puck with a crystal bond adhesive. The specimen was then affixed to the glass plate using a 1:1 epoxy–resin mixture. Before beginning the tests, an operational check and a preliminary experiment on fused silica were performed to verify the equipment’s functionality. The fused silica test confirmed an elastic modulus of 72 GPa, verifying the accuracy and readiness of the equipment. All nanoindentation experiments were conducted in a controlled laboratory environment to minimize the potential influence of external factors such as temperature and humidity on the elastic modulus measurements. The experimental setup maintained a constant room temperature of approximately 23 °C with a relative humidity level below 40%. These conditions were chosen to reduce the risk of thermal expansion or moisture absorption, particularly for the polyimide (PI) substrate, which can be sensitive to environmental changes. The fused silica reference sample tested prior to the experiments showed consistent elastic modulus values, confirming the stability and reliability of the setup.

Indentation depths were selected based on the thickness of the Al and Cu films. For the 0.5 μm films, indentation depths of 150 nm and 250 nm were applied; for the 1 μm films, depths of 300 nm and 500 nm were applied; and for the 1.5 μm films, depths of 500 nm and 750 nm were applied. Each specimen was subjected to a 5 × 5 grid of indentations, totaling 25 indentations, and five data points with high reproducibility were chosen for analysis.

A finite element analysis (FEA) was carried out using Abaqus (version 6.16) to model the indentation process. The indenter was modeled with a conical shape, and thin films were represented at thicknesses of 0.5, 1, and 1.5 μm for both Al and Cu on a 125 μm substrate. The thicknesses of 0.5, 1, and 1.5 μm were selected for the thin metal films to systematically evaluate the impact of film thickness on the measured elastic modulus and to ensure a range of thicknesses representative of practical applications in flexible electronics. The mesh was finely subdivided to create a high node density around the indentation site, with a total of 56,520 nodes and 55,068 elements in the substrate model. The CAX4R mesh type was used, and the indenter was treated as an analytical rigid body with a reference point for controlling its movement [27].

The materials modeled in the analysis included Al and Cu for the thin films and PI and Si for the substrates. Indentation depths were set to 30% and 50% of the film thickness for both Al and Cu films. The mechanical properties of the materials, including the elastic modulus and yield strength for the metal films and the elastic modulus for the substrates, were specified according to the values presented in Table 1.

## 4. Results and Discussions

**Figure 2:**
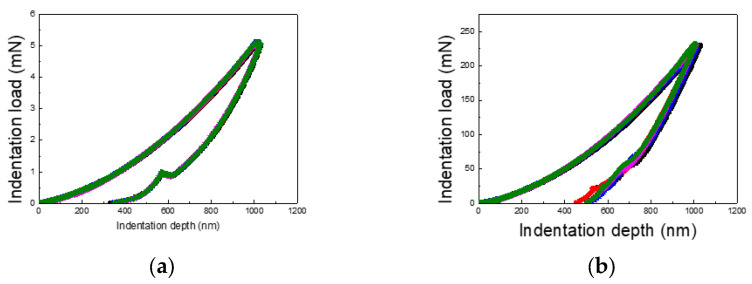
Load–displacement curves: (**a**) load–displacement curve for PI substrate and (**b**) load–displacement curve for Si substrate.

Figure 2 illustrates the load–displacement curves for the 1 µm indentation test conducted separately on PI and silicon substrates. Two notable differences are evident between the two materials. First, the slope of the unloading curve differs significantly. For the PI substrate, the unloading curve recovers to approximately 300 nm, while for the silicon substrate, the recovery reaches around 500 nm. This indicates a greater elastic recovery for the PI substrate compared to the silicon substrate. Second, the maximum indentation load varies significantly: approximately 5 mN for the PI substrate compared to 240 mN for the silicon substrate—a 48-fold increase. This demonstrates that the maximum load, even at the same indentation depth, is heavily influenced by the substrate material.

**Figure 3:**
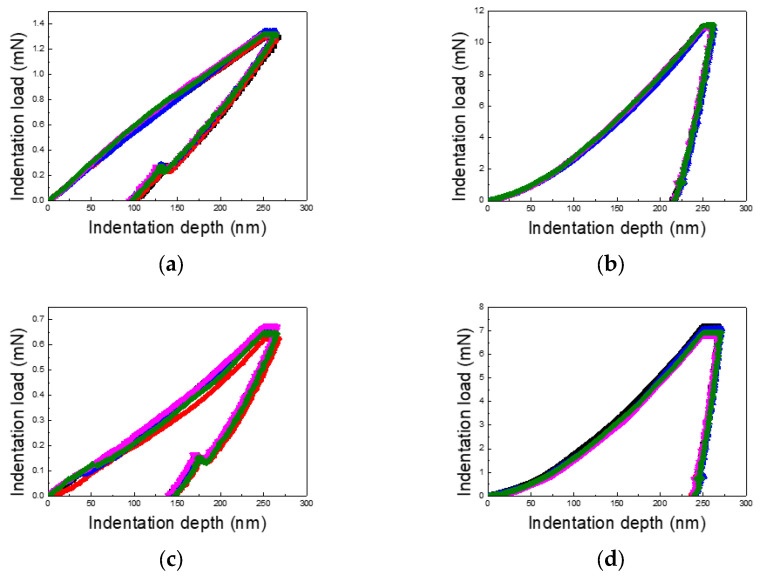
Load–displacement curves of Cu and Al thin films (film thickness of 0.5 μm and indentation depth of 50% of the film thickness): (**a**) Cu/PI, (**b**) Cu/Si, (**c**) Al/PI, and (**d**) Al/Si.

**Figure 4:**
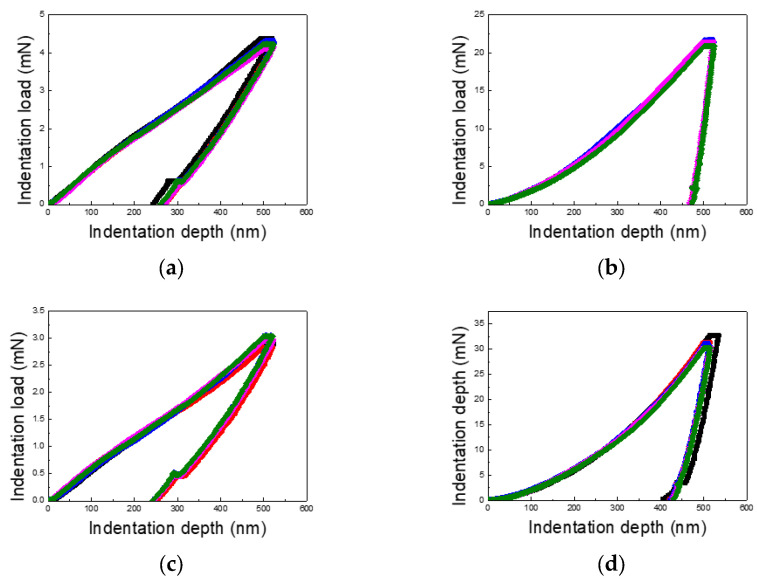
Load–displacement curves of Cu and Al thin films (film thickness of 1 μm and indentation depth of 50% of the film thickness): (**a**) Cu/PI, (**b**) Cu/Si, (**c**) Al/PI, and (**d**) Al/Si.

**Figure 5:**
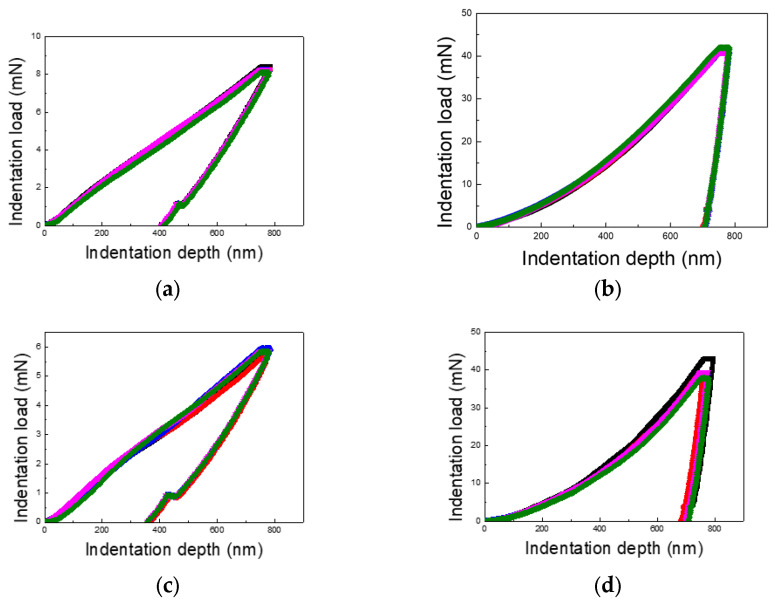
Load–displacement curves of Cu and Al thin films (film thickness of 1.5 μm and indentation depth of 50% of the film thickness): (**a**) Cu/PI, (**b**) Cu/Si, (**c**) Al/PI, and (**d**) Al/Si.

Figure 3, Figure 4 and Figure 5 present the load–displacement curves for the thin Cu and Al films obtained from nanoindentation experiments across varying indentation depths and differentiated by substrate type and film thickness. A consistent pattern emerges when comparing films on PI substrates to those on silicon wafer substrates: the unloading slopes for Cu and Al films on PI substrates are lower than those for films on silicon substrates, indicating a higher elastic recovery in films on PI substrates.

Additionally, the maximum indentation load for Cu films varies between 4–6 times, depending on the film thickness and indentation depth, while for Al films, the variation ranges from 4 to 10 times. This wide range underscores the significant role of film thickness and indentation depth in influencing the load response. The contrast between loading and unloading curves further highlights the substrate’s impact. Notably, for PI substrates, the loading curves deviate from the typical sharp-tip indentation curves described by Kick’s law. Instead, they exhibit a mid-curve inflection point, followed by a linear trend, likely due to the presence of two distinct layers with differing mechanical properties.

The unloading curves for the PI substrate samples display substantial elastic recovery, as seen in Figure 3, Figure 4 and Figure 5, with the final indentation depths being approximately 50% of the maximum depths. This highlights the strong elastic recovery characteristic of the PI substrate. In contrast, the silicon wafer samples exhibit more consistent loading and unloading curves without distinctive trends, reflecting the substrate’s uniform mechanical response across varying indentation depths.

**Figure 6:**
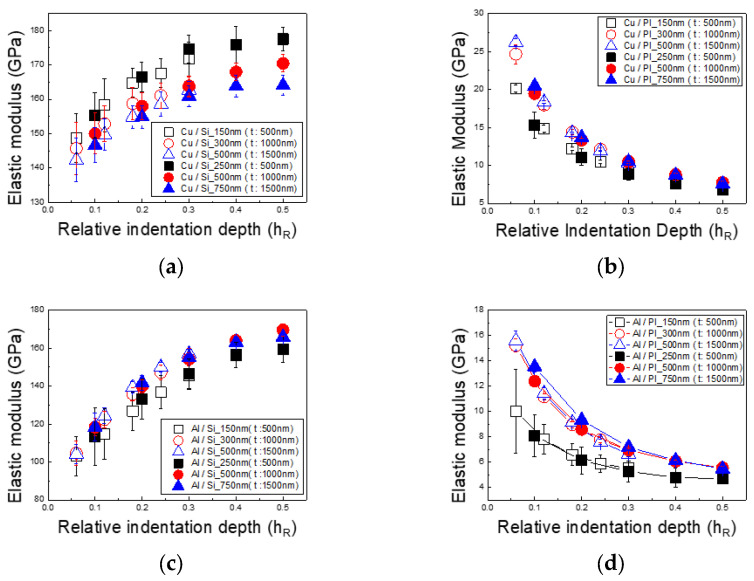
Elastic modulus based on substrate and film type and thickness: (**a**) Cu/Si, (**b**) Cu/PI, (**c**) Al/Si, and (**d**) Al/PI.

Figure 6 presents the elastic modulus of thin Cu and Al films on PI substrates and silicon wafer substrates as a function of indentation depth. Previous studies indicate that the typical elastic modulus of Cu is around 130 GPa, while that of Al is approximately 74 GPa [28].

Figure 6a shows the elastic modulus of a thin Cu film on a silicon substrate, which tends to increase with indentation depth. The modulus values range from a low of 140 GPa to a high of 180 GPa. Conversely, Figure 6b displays the elastic modulus of a thin Cu film on a PI substrate. For this sample, the elastic modulus decreases with an increasing indentation depth, ranging from a maximum of 21 GPa to a minimum of 7 GPa. Figure 6c shows the elastic modulus of a thin Al film on a silicon substrate. Similar to the Cu film on silicon, the Al film’s elastic modulus increases with indentation depth, with values ranging from 100 GPa to 170 GPa. Figure 6d shows the elastic modulus of a thin Al film on a PI substrate, which decreases with indentation depth, ranging from a maximum of 16 GPa to a minimum of 4 GPa. The elastic modulus of Cu and Al films on silicon substrates increased by approximately 50% and 130%, respectively, with indentation depth. On PI substrates, however, the modulus of elasticity for Cu and Al films decreased by about 85–95% and 80–95%, respectively. These trends indicate that as indentation depth increases, the measured elastic modulus of the thin film begins to approach that of the substrate material. For Cu and Al films on silicon substrates, the elastic modulus values are consistent with the properties of bulk Cu and Al, provided the 1/10 rule is applied. However, for Cu and Al films on PI substrates, the elastic modulus does not approach the typical values for bulk Cu and Al, even when the 1/10 rule is observed.

**Figure 7:**
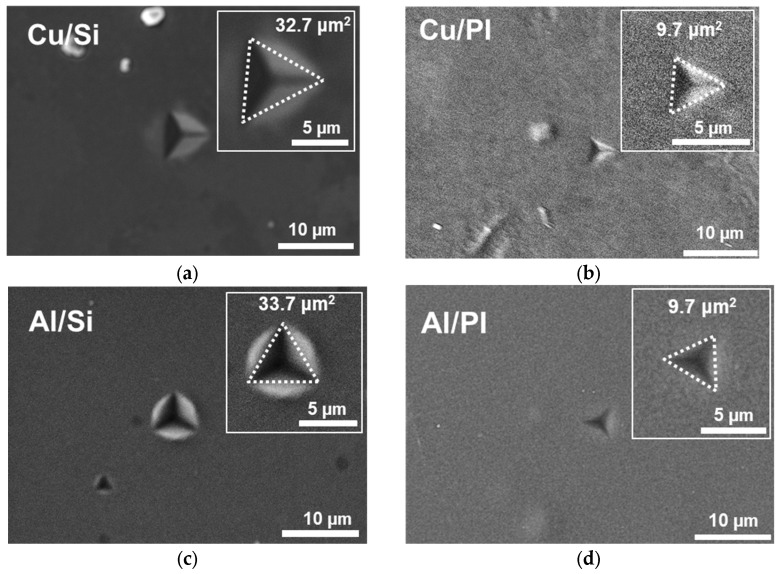
SEM images of the indentation of thin Cu and Al films on different substrate types: (**a**) Cu/Si, (**b**) Cu/PI, (**c**) AlSi, and (**d**) AlPI.

To investigate the effects of the silicon wafer and PI substrates on indentation behavior, the size of the indentations was measured using scanning electron microscopy (SEM). Figure 7 shows an SEM image of the indentation measurement. When comparing the indentation sizes of Cu and Al thin films on silicon wafer substrates with those on PI substrates, distinct differences are evident. On the silicon wafer substrate, the indentation size for Cu and Al films displays typical characteristics of metal indentation, such as pile-up. The contact area was determined by approximating the indentation mark as an equilateral triangle, connecting its vertices, and analyzing it using an image analysis program. As shown in the inset images of Figure 7, the measured areas were 33.7 μm^2^ for the Cu film and 32.7 μm^2^ for the Al film. In contrast, the indentations of Cu and Al films on PI substrates do not exhibit metal indentation features like pile-up. The indentation areas for both Cu and Al films on PI substrates were measured at 9.7 μm^2^, significantly smaller than those on the silicon wafer substrate. This difference in indentation size, as seen in the SEM images, is likely due to the influence of the substrate material. To further explore this hypothesis, a nanoindentation experiment was simulated through a finite element analysis (FEA), allowing for an examination of the stress field distribution and displacement behavior.

**Figure 8:**
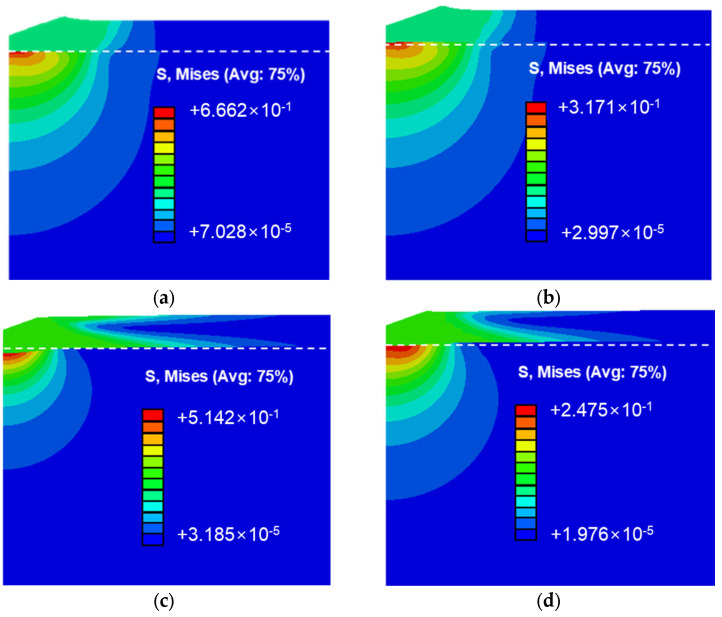
Finite element analysis results; stress field distribution due to substrate differences: (**a**) Cu/Si, (**b**) Al/Si, (**c**) Cu/PI, and (**d**) Al/PI.

Figure 8 illustrates the stress distribution in thin Cu and Al films on silicon and PI substrates. Figure 8a,b show the stress distribution for Cu and Al films on silicon substrates. The stress field forms in a concentric pattern, similar to the indentation of a single specimen. The maximum stress occurs at the interface where the thin film and the substrate meet directly beneath the indenter. Figure 8c,d display the stress distribution for Cu and Al films on PI substrates, where a similar trend is observed. However, the stress distribution patterns differ significantly between the substrates. On silicon substrates, the stress in the thin film closely mirrors the distribution in the underlying substrate in the horizontal direction. In contrast, on PI substrates, the stress in the substrate exhibits a concentric pattern, while the thin film shows a broad horizontal spread. This dispersion prevents a stress concentration directly beneath the indenter, extending deformation to distant regions and explaining the smaller indentation area on PI substrates.

**Figure 9:**
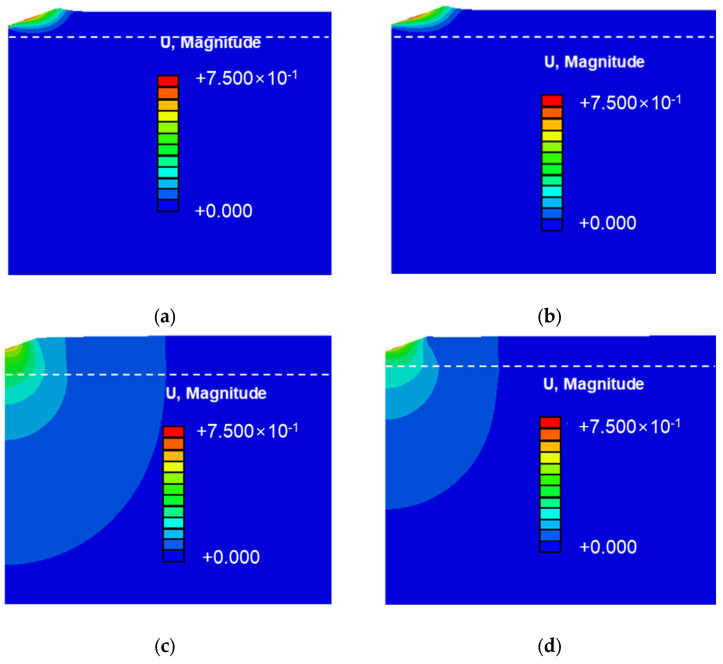
Finite element analysis results; displacement of thin Cu and Al films depending on substrate type: (**a**) Cu/Si, (**b**) Al/Si, (**c**) Cu/PI, and (**d**) Al/PI.

Figure 9 depicts the displacement patterns by substrate type. For silicon substrates, Cu and Al films show deformation localized near the indentation site. The high elastic modulus of silicon restricts deformation below the thin film, concentrating stress directly beneath the indenter and resulting in severe localized deformation, consistent with the larger indentation area. For PI substrates, deformation is more widespread, affecting both the indentation site and the surrounding substrate. The low elastic modulus of PI allows for excessive deformation, dispersing it over a broader area and reducing the indentation size. This distribution contributes to the underestimation of the elastic modulus on PI substrates. To accurately measure the elastic modulus of thin films while accounting for substrate effects, further analysis was performed using previously proposed models, as outlined in Equations (3)–(5) [20,21,26].

As mentioned in Section 2, numerous models have been proposed to improve accuracy in determining the elastic modulus [22,23,25,26]. However, these models are challenging to apply directly, as they require knowledge of the experimental constant α used in the weighting factor. To address this limitation, this study introduces a new approach by replacing the weighting factor $e^{-\frac{\alpha (t-h)}{a}}$ in Equation (5) with a variable called X, creating a revised weighting model.

(6) $$\frac{1}{E_{r}}=\frac{1-{v_{i}}^{2}}{E_{i}}+\frac{1-{v_{f}}^{2}}{E_{f}}\left(1-X\right)+\frac{1-{v_{s}}^{2}}{E_{s}}\left(X\right)$$

**Figure 10:**
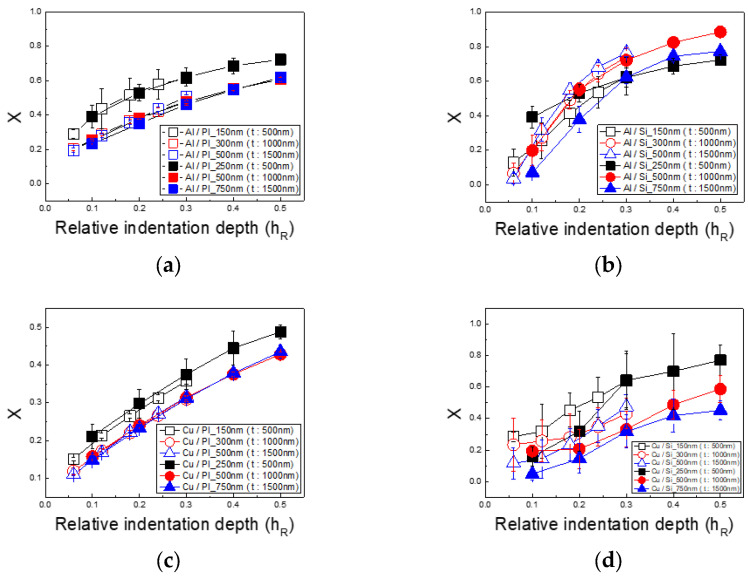
Weighting factor of substrates based on substrate and film type: (**a**) Al/PI, (**b**) Al/Si, (**c**) Cu/PI, and (**d**) Cu/Si.

Figure 10 shows the results of calculating the weighting factor X for PI to correct the underestimation of the elastic modulus in thin PI films. Since all variables in Equation (7) are known except X, the value of X can be derived. As referenced above [22,23,25,26], X can be defined as the substrate’s contribution to *E*_*r*_, with X values increasing as the indentation depth grows. This reflects the fact that with a greater penetration depth, the stress field extends from the thin film into the substrate, indicating that substrate deformation increasingly impacts the indentation load.

**Figure 11:**
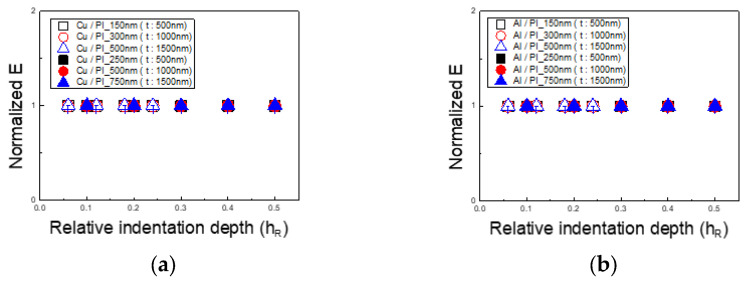
The results for calibrating the modulus of elasticity of the thin films: (**a**) Cu/PI and (**b**) Al/PI.

Based on the calculated X values, Figure 11 presents the corrected elastic modulus for thin films on PI substrates. The results, normalized by dividing the PI substrate’s elastic modulus by that of the silicon substrate following the 1/10 rule, converge to 1, indicating successful calibration. For both the Cu/PI and Al/PI specimens, the error across all indentation depths was within 2%, confirming the effective application of the X-based weighting model methodology for accurate elastic modulus measurements.

## 5. Conclusions

This study introduced an advanced model with weighting factor X to correct the underestimation of the elastic modulus of thin metal films on flexible substrates. The proposed model successfully accounted for the influence of substrate properties, as validated through nanoindentation experiments and a finite element analysis (FEA). The results show significant improvements in measurement accuracy, with errors reduced to within 2%, making the model a reliable tool for characterizing thin films on flexible substrates. These findings have practical implications for the design and durability evaluation of flexible electronics. While the proposed model demonstrates significant improvements, its applicability to other types of flexible substrates, such as elastomers or hybrid multilayer films, has not been fully explored. Substrates with a more complex mechanical behavior or time-dependent properties (e.g., viscoelasticity) may require additional modifications to the model. Furthermore, the current study focused primarily on room-temperature conditions. The integration of thermal effects, such as changes in mechanical properties due to thermal expansion or temperature-dependent modulus variations, represents a critical direction for future work. Such advancements would further enhance the model’s utility for real-world applications where flexible devices are exposed to varying environmental conditions.

## Figures and Tables

**Table 1:** Mechanical properties of thin films and substrates used in FE analysis.

	Silicon Wafer	Polyimide	Cu	Al
Elastic modulus (GPa)	170	3.61	130	70.4
Yield strength (MPa)	-	-	262	124
Poisson’s ratio	0.3	0.3	0.3	0.3

## Data Availability

The original contributions presented in this study are included in the article. Further inquiries can be directed to the corresponding author.
